# OCCUPATIONAL HEALTH: On the Job with Solar PV

**DOI:** 10.1289/ehp.118-a19

**Published:** 2010-01

**Authors:** David A. Taylor

**Affiliations:** **David A. Taylor** writes for *The Washington Post* and *Smithsonian* and is author of *Ginseng, the Divine Root*, about the science and subculture surrounding the medicinal plant. He teaches science writing at The Writer’s Center in Maryland

Solar photovoltaic (PV) energy is the world’s fastest-growing alternative energy technology. The cost of installed solar PV fell by more than 30% over the past decade, according to *Tracking the Sun II: The Installed Cost of Photovoltaics in the U.S. from 1998–2008*, an October 2009 study from Lawrence Berkeley National Laboratory. Solar PV is also by most accounts one of the cleanest renewable energy sources.

But the manufacture of solar PV materials involves compounds with health and environmental risks, issues the burgeoning industry is grappling with. “New solar PV technologies are increasing cell efficiency and lowering costs, but many of these use extremely toxic materials or materials with unknown health and environmental risks (including new nanomaterials and processes),” cautioned the authors of *Toward a Just and Sustainable Solar Energy Industry*, a January 2009 white paper issued by the Silicon Valley Toxics Coalition.

The main commercial technologies use either silicon wafers in rigid panels (for example, installed on residential rooftops) or materials coated with thin PV films, which are used in applications such as wall coatings and roof tiles. Silicon tetrachloride (SiCl_4_) is a volatile intermediate form of silicon used and produced in manufacturing silicon PV wafers, while cadmium telluride (CdTe) and amorphous silicon (a-Si) are used to produce thin films. Although cadmium is a known carcinogen, according to the National Toxicology Program, the toxicity of CdTe is less than that of the parent compound.

Manufacturing both cadmium- and a-Si–based PV materials typically involves depositing ångström-thick layers of gases such as arsine, phosphine, and silane onto a substrate. These gases are considered extremely hazardous, highly toxic, or pyrophoric (meaning they combust as soon as they hit the air). The highly pressurized gases used for creating thin PV films pose the main occupational dangers, according to Vasilis Fthenakis, senior scientist at Brookhaven National Laboratory in Upton, New York, who outlined the occupational hazards of solar PV manufacturing in the 15 March 2008 issue of *Environmental Science and Technology*. Fthenakis noted in his report that safety protocols for these compounds are well established for the industry, but “for newcomers in photovoltaics, there is a learning curve.”

Fthenakis also wrote that the most efficient strategy to reduce the hazards associated with PV manufacture is simply to choose technologies that don’t require large quantities of hazardous gases. “In general, thin-film photovoltaics require less energy in their manufacturing than crystalline Si photovoltaics,” he wrote. This translates to lower emissions of toxics such as heavy metals, sulfur oxides, nitrogen oxides, particulate matter, and carbon dioxide.

Typically in U.S. solar plants, as in electronics manufacturing, each phase of the assembly and spray of the PV surface is contained in “glovebox” isolators and modular cleanrooms. In the wake of incidents where SiCl_4_ was released improperly, the industry has been adding controls for safety, says Fthenakis. And a working group of SEMI, a global industry association serving the PV and other manufacturing sectors, is developing an industry safety standard.

Enforcement of occupational and environmental standards will remain in the hands of individual manufacturers, however. A 9 March 2008 *Washington Post* report by Ariana Eunjung Cha described health problems experienced by villagers in Henan Province, China, when a solar PV manufacturer dumped SiCl_4_ near farmers’ fields. Villagers complained that exhaust from the factory’s chimneys stung their eyes and nasal passages, and the white powder dumped on fields—which produces hydrochloric acid and the other acid fumes when it meets air—caused crops to wilt. A U.S. safety engineer who requested anonymity points out, however, that “the incident in China was a failure of the local authorities to enforce standards that they have in effect, not a failure of the PV industry to recognize and address hazards.”

Jason Sharpe, chief operating officer at Namasté Solar, a Colorado-based company that installs silicon PV panels, says most manufacturing occurs outside the United States—China, in fact, is now the world’s largest producer of solar PV materials. When Sharpe tried to learn more about details of standards used by their suppliers, he encountered obstacles. “There’s not a lot of transparency,” he says. “You don’t know a lot of the details of what your manufacturers do.”

Namasté Solar decided to deal only with manufacturers that publish corporate responsibility reports, operate predominantly in North America, and have demonstrated they follow the strictest environmental and occupational safety guidelines, he says. These included San Jose–based SunPower Corporation.

The manufacturing process “is very similar to a semiconductor process” except it involves fewer steps, explains Julie Blunden, SunPower’s VP for public policy and corporate communications. “We’re fortunate to have benefitted from a long line of improvements in the semiconductor industry.” Depending on plant location, SunPower follows the strictest of three protocols: U.S. federal rules, World Bank rules, or those of the local jurisdiction.

Despite the potential risks associated with PV production, Sharpe and Fthenakis both emphasize it’s important to look at the big picture when considering the sustainability of PV: overall, lifetime emissions of greenhouse gases and other pollutants are much lower for PV technologies than for conventional fossil fuel energy sources; Fthenakis estimates that replacing grid electricity with central PV systems could avert at least 89% of air emissions associated with conventional electricity generation.

Realistically, continuing pressure to decrease costs could make for less-than-transparent procedures. On the other hand, says Sharpe, “Because solar PV modules are rapidly becoming a commodity, manufacturers are striving to find ways to differentiate themselves.” Highlighting adherence to occupational and environmental health standards is, he says, “a great opportunity.”

## Figures and Tables

**Figure:**
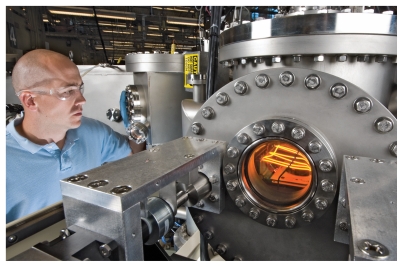
A glowing 2,000°C filament decomposes silane and hydrogen gases to deposit thin films of a-Si on the surface of a heated substrate.

